# Design and preclinical evaluation of a novel apelin-based PET radiotracer targeting APJ receptor for molecular imaging of angiogenesis

**DOI:** 10.1007/s10456-023-09875-8

**Published:** 2023-03-27

**Authors:** Béatrice Louis, Vincent Nail, Oriane Nachar, Ahlem Bouhlel, Anaïs Moyon, Laure Balasse, Stéphanie Simoncini, Adrien Chabert, Samantha Fernandez, Pauline Brige, Guillaume Hache, Aura Tintaru, Clément Morgat, Françoise Dignat-George, Philippe Garrigue, Benjamin Guillet

**Affiliations:** 1 Aix Marseille Univ, INSERM, INRAE, C2VN, Marseille, France; 2 Aix Marseille Univ, CNRS, CERIMED, Marseille, France; 3 Assistance Publique - Hôpitaux de Marseille, Pôle Pharmacie, Radiopharmacie, Marseille, France; 4 Aix Marseille Univ, LIIE, Marseille, France; 5 Aix Marseille Univ, CNRS, CINaM, Marseille, France; 6 Univ. Bordeaux, CNRS, INCIA, UMR 5287, 33000 Bordeaux, France; 7 Nuclear Medicine Department, University Hospital of Bordeaux, 33000 Bordeaux, France

**Keywords:** Angiogenesis, Apelin, APJ, PET imaging, Theranostics

## Abstract

APJ has been extensively described in the pathophysiology of angiogenesis and cell proliferation. The prognostic value of APJ overexpression in many diseases is now established. This study aimed to design a PET radiotracer that specifically binds to APJ. Apelin-F13A-NODAGA (AP747) was synthesized and radiolabeled with gallium-68 ([^68^Ga]Ga-AP747). Radiolabeling purity was excellent (> 95%) and stable up to 2 h. Affinity constant of [^67^Ga]Ga-AP747 was measured on APJ-overexpressing colon adenocarcinoma cells and was in nanomolar range. Specificity of [^68^Ga]Ga-AP747 for APJ was evaluated in vitro by autoradiography and in vivo by small animal PET/CT in both colon adenocarcinoma mouse model and Matrigel plug mouse model. Dynamic of [^68^Ga]Ga-AP747 PET/CT biodistributions was realized on healthy mice and pigs for two hours, and quantification of signal in organs showed a suitable pharmacokinetic profile for PET imaging, largely excreted by urinary route. Matrigel mice and hindlimb ischemic mice were submitted to a 21-day longitudinal follow-up with [^68^Ga]Ga-AP747 and [^68^Ga]Ga-RGD_2_ small animal PET/CT. [^68^Ga]Ga-AP747 PET signal in Matrigel was significantly more intense than that of [^68^Ga]Ga-RGD_2_. Revascularization of the ischemic hind limb was followed by LASER Doppler. In the hindlimb, [^68^Ga]Ga-AP747 PET signal was more than twice higher than that of [^68^Ga]Ga-RGD_2_ on day 7, and significantly superior over the 21-day follow-up. A significant, positive correlation was found between the [^68^Ga]Ga-AP747 PET signal on day 7 and late hindlimb perfusion on day 21. We developed a new PET radiotracer that specifically binds to APJ, [^68^Ga]Ga-AP747 that showed more efficient imaging properties than the most clinically advanced tracer of angiogenesis, [^68^Ga]Ga-RGD_2_.

## Introduction

Angiogenesis is the adaptive process allowing new vessels formation from pre-existing ones [1]. In pathophysiological conditions such as vascular, oncologic, or inflammatory diseases, angiogenesis can be up- or down-regulated depending on the complex balance of context-related factors expression. Angiogenesis, therefore, represents a relevant therapeutic target for preservation, regeneration, and functional recovery during and after ischemic injury [2], or its inhibition to counteract tumor proliferation and dissemination [3].

Tissue angiogenesis monitoring at the whole-body scale is essential to guide and support angiogenesis-targeting therapeutic strategies, given the elevated inter- and intra-individual heterogeneity [4]. Such a tool has not been validated at the clinical stage yet. Non-invasive molecular imaging of angiogenesis may represent a valuable way as a companion tool to a given pro- or anti-angiogenic therapy [1, 5, 6]. Molecular imaging enables the assessment of the expression of molecular targets with the highest sensitivity [7–13]. Since the early 2000s, several molecular targets have been evaluated for angiogenesis imaging. Numerous molecular targets have been documented for molecular imaging of angiogenesis [14] above which Vascular Endothelial Growth Factor/Vascular Endothelial Growth Factor Receptors (VEGF/VEGFRs) and α_v_β_3_ integrins represent the most studied [13, 15]. Radiotracers targeting soluble VEGF and then VEGF receptors or integrin-targeting Arginylglycylaspartic acid (RGD)-based imaging agents, in line with their involvements in angiogenesis have been largely evaluated but not led to any approved molecular imaging probe yet despite many optimizations for pharmacokinetics, selectivity, and affinity improvement [16–19]. Recent reports focused on targeting aminopeptidase (CD13), metalloproteases, and angiomotin; however, their specificity and interest at the clinical stage remain to be evaluated [20–22]. For the lack of any more clinically advanced radiotracer, [^68^Ga]Ga-RGD and its variants are often considered as the reference radiotracer for molecular imaging of angiogenesis [23]. However, after more than 20 years of development, there is still no related marketing authorization, probably due to the choice of the molecular target.

In this context, we identified the apelin/APJ system as a relevant molecular target for angiogenesis imaging because of its implication in blood vessels formation by sprouting [24, 25]. The APJ receptor also known as APLNR and AGTRL1 is a class A G-protein-coupled receptor. Apelin is an endogenous ligand of this receptor. The Apelin/APJ system is described as implicated in different pathologies [26–29]. High levels of APJ mRNA and protein are found respectively in endothelial cells through vessel formation [30] and on sprouting vessels of hypoxic tissues and tumor-associated endothelium [31, 32]. Recently, several APJ agonists and antagonists [33] were designed and modulation of the apelin/APJ emerged as a potential therapeutic approach [34–36]. Accordingly, we hypothesized the added value of developing a specific APJ-targeting radiotracer based on apelin-F13A based on its nanomolar affinity for APJ [33]. This work aimed at designing, developing, and characterizing a [^68^ Ga]Ga-radiolabeled apelin-F13A—codenamed [^68^ Ga]Ga-AP747—to evaluate its interest as a novel agent for PET imaging of angiogenesis and APJ expression quantification.

## Materials and methods

### AP747 synthesis

One milligram of apelin-F13A (Merck Millipore, Burlington, USA) was added to 10 equivalents (14 mg) of NODA-GA-NHS ester (CheMatech, Dijon, France) solubilized in 700 µL of 0.2 mol/L bicarbonate buffer. The conjugate was then transferred to a tC18 cartridge (Sep-Pak, Waters, Milford, USA) and then eluted with 500 µL of HPLC-grade absolute ethanol (VWR, Radnor, USA). The solvent was evaporated at room temperature and 500µL HPLC-grade water (VWR, Radnor, USA) were added. Aliquots (10 µL) were stored at – 20 °C.

### High-resolution mass spectrometry (HRMS) characterization

The characterization of the resulting AP747 was performed by HRMS, using a Waters SYNAPT G2 HDMS II (Manchester, United Kingdom) equipped with an electrospray source, operated under positive ionization mode [ESI—( +)], and a quadrupole/time of flight analyzer (QTOF). Capillary voltage was fixed at 2.8 kV, and the declustering potential was optimized at 30 V; N_2_ at 100 L/h flow and 35 °C was used as desolvation gas. The samples were diluted (1/100, v/v) in methanol doped with 1% formic acid and infused at 10 µL/min flow rate into the ESI source. The m/z values were calibrated by Mass Lock procedure using a methanolic solution of positively charged clusters of sodium acetate. The experimental data were acquired and processed using MassLynx 4.1 (Waters, Milford, USA).

### Radiolabeling of AP747 and RGD_2_ with gallium-68

Fifty microliters of 4 mol/L ammonium acetate buffer (pH 7.4) were added to a 10 µL AP747 sample (1 μg/μL) or a 10 µL NODAGA-RGD dimer acetate sample (1 μg/μL, ABX, Radeberg, Germany). 500 µL of [^68^Ga]GaCl_3_ (200.6 ± 40.9 MBq/500 µL) were eluted from a commercial TiO_2_-based [^68^Ge]Ge/[^68^Ga]Ga generator (Galliapharm, Eckert & Ziegler Berlin, Germany) using 0.1 mol/L HCl and added to the reactor.

Final pH of the mixture was 6.0. Radiochemical purity (RCP) was determined by radio-thin-layer chromatography (radio-TLC) on a miniGITA radio-TLC scanner detector (Elysia-Raytest, Straubenhardt, Germany) using iTLC-SG plate as solid phase (Agilent, Les Ulis, France) and a mixture of 1 mol/L aqueous ammonium acetate solution and methanol 1:1 (v/v) as mobile phase 1 (free [^68^Ga]GaCl_3_ Rf = 0; [^68^Ga]Ga-AP747 Rf = 0.5; [^68^Ga]Ga-RGD_2_ Rf = 1) and trisodium citrate 0,1 mol/L pH = 5 as mobile phase 2 ([^68^Ga]Ga-AP747 or [^68^Ga]Ga-RGD_2_ Rf = 0; free [^68^Ga]GaCl_3_ Rf = 1). Radiochemical purity was also determined by radio-high pressure liquid chromatography (radio-HPLC) with an Ultimate 3000 pump (Dionex, ThermoFisher, Waltham, USA) using a reversed-phase C18-column (Luna LC Column 150 × 4.6 mm, Phenomenex, Torrance, USA), an in-line radioactivity detector (Gabi, Elysia-Raytest, Straubenhardt, Germany) and a gradient of water/acetonitrile with 0.1% trifluoroacetic acid (TFA) from 100/0 (v/v) to 0/100 (v/v) for 15 min at a flow rate of 2.5 mL/min (free [^68^ Ga]GaCl_3_ retention time: 1.0 min; [^68^Ga]Ga-RGD_2_ retention time = 3.5 min; [^68^Ga]Ga-AP747 retention time: 4.0 min). Radio-HPLC chromatograms were interpreted using Chromeleon software (ThermoFisher Scientific, Waltham, USA). The evaluation of [^68^Ga]Ga-AP747 radiochemical stability was performed as previously described [37] after incubating 100 µL of the radiotracer in 400 µL of physiological saline or in 400 µL of human serum. The radiochemical purity was checked 60 and 120 min after radiosynthesis by radio-TLC, at room temperature and at 37 °C.

### Radiolabeling of AP747 with gallium-67

Gallium-67 was obtained from [^67^Ga]Ga-citrate (200 MBq, Curium, Paris, France) and converted into [^67^Ga]GaCl_3_ using two Light silica Sep-Pak (Waters, Milford, USA). Briefly, [^67^Ga]Ga-citrate was charged on the cartridges and then eluted using 1 mL 0.1 mol/L HCl (KT720P, Rotem Industries, Dimona, Israël) in form of [^67^Ga]GaCl_3_ and subsequently used for radiolabeling. The final product was formulated in 3 mL of Phosphate Buffer Saline (PBS, Eurobio-scientific, Les Ulis, France). This solution was then added to AP747 (1 μg/μL). The RCP was determined by radio-TLC and was performed using iTLC-SG plate as solid phase (Agilent, Les Ulis, France) and trisodium citrate 0.1 mol/L as mobile phase ([^67^Ga]Ga-AP747 Rf = 0, [^67^Ga]GaCl_3_ Rf ≥ 0.8, [^67^Ga]Ga-NODAGA Rf ≥ 0.8).

### [^67^Ga]Ga-AP747 lipophilicity

Determination of logD value was realized by the shake-flask method [38]. Briefly, 50 µL of [^67^Ga]Ga-AP747 was added to a 1 mL solution of octanol and physiological serum (1:1). This solution was stirred and vortexed for two minutes and centrifugated (100 g, 5 min). Three 100 µL samples of each phase were collected, and the respective activity was measured using a gamma counter (Wizard 2480, Perkin-Elmer, Waltham, USA).

### Cell culture

Human colon adenocarcinoma T84 cell line (RRID:CVCL_0555) was cultivated in filtrated DMEM-F12 (Gibco, ThermoFisher Scientific, Waltham, USA)/Glutamax (Gibco, ThermoFisher Scientific, Waltham, USA) medium. Human umbilical vein endothelial cells (HUVECs, gratefully from the Cell Therapy Laboratory, La Conception University Hospital, AP-HM, Marseille, France) were cultivated in filtrated Endothelial Cell Growth Medium MV (EGM-2 SupplementMix, Promocell, Heidelberg, Germany). Both media were supplemented with 10% decomplemented fetal bovine serum and 1% antibiotic–antimycotic mix (penicillin–streptomycin). Cell lines were maintained in a humidified 5% CO_2_ incubator at 37 °C. HUVECs activation was obtained by overnight incubation with Tumor Necrosis Factor alpha (TNF-alpha, 10 ng/mL, Euromedex, Souffelweyersheim, France).

### APJ cell expression

APJ expression was evaluated by Western Blot. Cell lysates of human colon adenocarcinoma T84 cells and human umbilical vein endothelial cells (HUVECs) in TNF-activated or baseline (PBS) conditions were loaded on polyacrylamide gel (NuPAGE, 4–12%, Invitrogen, Waltham, USA). After migration, proteins were transferred to a nitrocellulose membrane and checked by Rouge-Ponceau. The membrane was saturated [Tris-buffered saline Tween 20% (TBST); bovine serum albumin (BSA) 3%], then an anti-APJ antibody (1 µg/mL, 5H5L9, rabbit monoclonal Invitrogen, Waltham, USA) was added overnight, under agitation at 4 °C. After three TBST washes, a secondary goat anti-rabbit horseradish peroxidase (HRP)-tagged antibody (1/2000, #31460, ThermoFisher, Waltham, USA) was added for one hour. Revelation was achieved with an enhanced chemiluminescent (ECL) kit (#32106, ThermoFisher, Waltham, USA) with an incubation of 5 min at room temperature. Membrane image acquisitions were realized through a Gbox system (Syngene, Cambridge, UK) with an exposure time of 1 min. After 10 min of stripping and three washes, the membrane was saturated [Tris-buffered saline Tween 20% (TBST); bovine serum albumin (BSA) 3%] and incubated with a GADPH antibody [#2118, (14C10) rabbit monoclonal, Cell Signaling Technologies, USA] at 4 °C overnight under agitation as the anti-APJ antibody. After three TBST washes, a secondary goat-anti-rabbit HRP-tagged antibody (1/2000, #31460, ThermoFisher, Waltham, USA) was added for half an hour. Revelation and membrane image acquisition were completed as described above with an exposure time of 30 s. The optical density of each band was measured using a specific software (GeneTools, Syngene, Cambridge, UK). APJ expression evaluation was normalized by division of GAPDH expression as protein load control.

### In vitro saturation binding assay

T84 cells were seeded at a 250.10^3^ cells per well density in 24-well plates (Corning, Corning, USA) and incubated overnight with complete medium. Plates were set on ice 30 min before the beginning of the experiment. [^67^Ga]Ga-AP747 was then added to the medium at a 0.1, 1, 10, 100, or 250 nmol/L concentration and cells were incubated for 2 h at 4 °C, in quadruplicate (*n* = 3). Incubation was stopped by removing the medium and washing cells twice with ice-cold PBS (Eurobio-scientific, Les Ulis, France). Finally, cells were treated with 1 mol/L NaOH, and the activity was measured using a gamma counter (Wizard 2480, Perkin-Elmer Waltham, USA). In order to assess non-specific affinity, an excess of non-radioactive apelin-F13A (final concentration 1 µmol/L) was added to selected wells.

### In vitro*** evaluation of [***^***68***^***Ga]Ga-AP747 specificity***

T84 cells were seeded at a 1.10^6^ cells per well density in 24-well plates (Corning, Corning, USA) and incubated overnight with a complete medium. [^68^Ga]Ga-AP747 (1.7 MBq/10 µL) was added to each well. In 6 wells, a large excess (500 µg/500 µL) of apelin-F13A (Phoenix Pharmaceuticals, Burlingame, USA) was added to T84 cells 10 min before incubation with [^68^Ga]Ga-AP747 (triplicate, *n* = 6). After a 1 h incubation, the medium was removed and the cells were washed 3 times with PBS (Eurobio-scientific, Les Ulis, France) and their viability assessed. The wells were measured for [^68^Ga]Ga-AP747 signal by autoradiography using a phosphor-based Cyclone autoradiograph (Perkin-Elmer, Waltham, USA). The background signal was measured through [^68^Ga]Ga-AP747 activity in wells without cells.

### Animal experiments

All procedures involving animals were approved by the Institution’s Animal Care and Use Committee (CE71, Aix-Marseille Université, projects #15790, #32157, #31843), conducted according to the 2010/63/EU European Union Directive and the ARRIVE guidelines 2.0 [39]. Mice were housed in enriched cages and placed in a temperature- and hygrometry-controlled room with daily monitoring, fed with water, and commercial diet ad libitum. Pigs were housed in enriched boxes with daily monitoring with water ad libitum and a commercial diet adapted with their nutritional requirements.

### [^68^Ga]Ga-AP747 biodistribution in healthy mice

Nine-week-old male Swiss mice (Janvier Labs, *n* = 3) were injected in the lateral caudal vein with [^68^Ga]Ga-AP747 (4.45 ± 0.32 MBq/70 µL), and small animal PET images were continuously acquired right after, up to 2 h post-injection. The quantified PET signal in organs was presented as mean ± SD percentage of the decay-corrected injected dose (%ID). Acquisition of small animal dynamic PET/CT was performed for 120 min on a NanoScan PET/CT camera (Mediso, Budapest, Hungary) under 2% isoflurane in medical air anesthesia [PET parameters: numbers of iterations: 4, coincidence: 1–3, field of view (FOV): 10 cm]. CT parameters were fixed at 35 kV voltage, 300 ms exposure at medium zoom, acquired by semi-circular method on the same FOV as PET. CT attenuation-corrected reconstruction was performed using Nucline software (Mediso, Budapest, Hungary) on the following time frames: 0–5 min, 6–10 min, 11–15 min, 16–20 min, 21–25 min, 26–30 min, 31–45 min, 46–60 min, 61–75 min, 76–90 min, 91–105 min, and 106–120 min. Quantitative volume-of-interest (VOI) analysis of the small animal PET images was CT-based manually performed on attenuation- and decay-corrected PET images using VivoQuant software (v.3.5, InVicro, Boston, USA).

Three 9-week-old Swiss male mice were injected in the lateral caudal vein with [^68^Ga]Ga-AP747 (4.02 ± 0.16 MBq/70 µL) and maintained under isoflurane anesthesia (2%) for two hours. Blood was collected at 2, 5, 10, 15, 20, 30, 45, 60, 75, 90, 105, and 120 min post-injection and gamma counted with decay correction. Plasmatic half-life (t_1/2_) was estimated by nonlinear regression. At two hours post-injection, mice were euthanatized and the main organs (heart, liver, lungs, muscle, brain, spleen, intestines, bone, pancreas, and kidneys) were collected, washed in PBS, weighted, and gamma counted. Results were decay corrected and expressed as percentage of injected dose corrected by organ weight (%ID/g).

### [^68^Ga]Ga-AP747 biodistribution in healthy swine

Six-month-old Pietrain pigs (*n* = 3, Blossin, Aubagne, France) were injected with [^68^Ga]Ga-AP747 (85 ± 30 MBq/5 mL in 0.9% NaCl) under 2% sevoflurane in medical air anesthesia. A series of six 15-min-long, static PET/CT acquisitions (Discovery PET/CT 710, GE Healthcare, Chicago, Illinois, USA) was started 15 min after the injection of [^68^Ga]Ga-AP747 and repeated at 45 min, 75 min, 90 min, 105 min, and 120 min post-injection (PET parameters: numbers of iterations: 4, coincidence: 1–1, FOV: 50 cm, reconstruction type: VPHD; CT parameters: 120 kV, 220 mA, slice thickness: 3,75 mm, number of pictures: 47, detector width: 40 mm). Attenuation-corrected images were reconstructed using ADW software v4.6 (GE Healthcare, Chicago, Illinois, USA), and PET signal quantifications were performed on CT-based manually drawn volumes of interest (VOIs) using VivoQuant software v.3.5 (InVicro, Boston, MA, USA). Results were decay corrected and expressed as mean ± sd percentage of the injected dose (%ID).

### Human colon adenocarcinoma mouse model

Human colon adenocarcinoma xenografts were established by subcutaneous dorsal injections of 1.10^6^ T84 cells (100 µL, PBS) to 6-week-old male Swiss nude mice (Charles River, Saint-Germain-Nuelles, France, *n* = 3) under 2% isoflurane anesthesia.

### Matrigel plug mouse model

Matrigel plugs were established by subcutaneous dorsal injections of 300 µL Matrigel (Dutscher, Bernolsheim, France) supplemented with 10% fetal bovine serum, to 9-week-old male Swiss mice (Janvier Labs, Le Genest-Saint-Isle, France, *n* = 12) under 2% isoflurane anesthesia. Seven Matrigel mice underwent small animal PET follow-up, and five others were involved in the in vivo specificity study as described below.

### In vivo specificity of [^68^Ga]Ga-AP747 PET signal

Mice bearing ectopic colon adenocarcinoma xenograft (*n* = 3, 1370 ± 167.3 mm^3^) or Matrigel plug (*n* = 5, 635 ± 146 mm^3^) were injected in the lateral caudal vein with [^68^Ga]Ga-AP747 (5.14 ± 0.60 MBq/80 µL) and underwent small animal PET imaging acquired 1 h p.i. followed by a CT scan. Small animal PET imaging acquisition lasted 20 min (number of iterations: 4, coincidence: 1–3) using a field of view (FOV) of 10 cm. CT parameters were fixed at 35 kV voltage, 300 ms exposure at medium zoom, acquired by semi-circular method on the same FOV as PET. CT attenuation-corrected reconstruction was performed using Nucline software (Mediso, Budapest, Hungary). The day after, the mice received an intravenous injection of a 100X excess of unconjugated peptide (apelin-F13A, 100 µg/100 µL) 30 min before the intravenous injection of 5.5 ± 0.20 MBq/80 µL [^68^Ga]Ga-AP747. PET images were acquired 1 h after [^68^Ga]Ga-AP747 injection. Tissue uptake values were expressed as a mean target-to-background PET signal ratio (TBRmean) with background represented by the left gastrocnemius muscle.

### Hindlimb ischemia mouse model

Unilateral hindlimb ischemia (HLI) was induced on 9-week-old female Swiss mice (Janvier Labs, *n* = 8) after femoral artery excision under 2% isoflurane anesthesia as previously described [40]. LASER Doppler perfusion imaging (Perimed, Craponne, France) was used to assess revascularization from day 0 to day 21 after surgery. Perfusion measurements were expressed as an ischemic-to-contralateral ratio of hind limb blood flow normalized to the day of surgery.

### In vivo longitudinal study using [^68^^68^Ga]Ga-AP747 PET compared with [Ga]Ga-RGD PET_2_

**Fig. 1:**
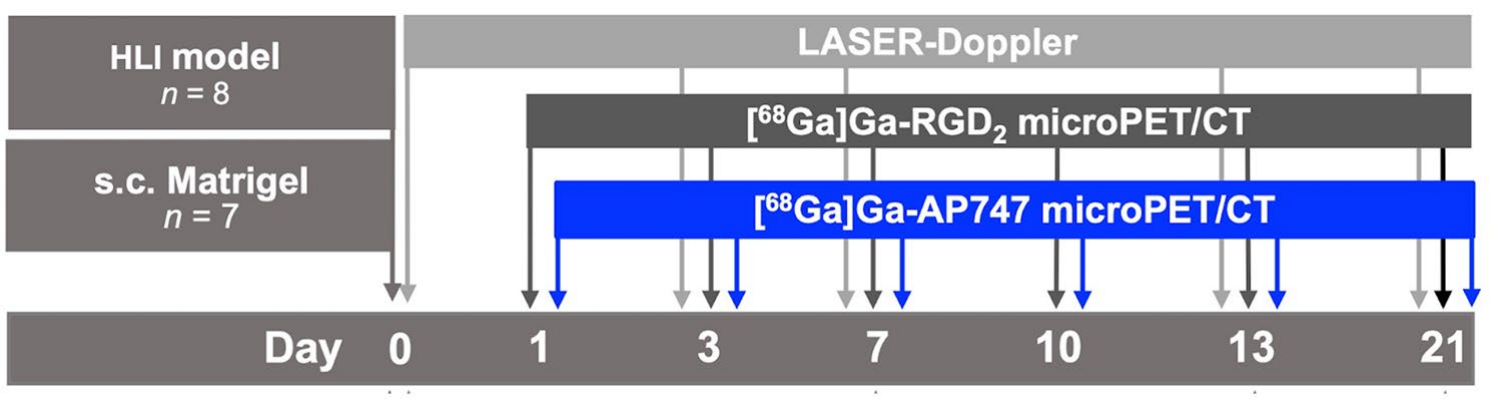
In vivo longitudinal study using [^68^Ga]Ga-AP747 PET compared with [^68^Ga]Ga-RGD_2_ PET on Matrigel and HLI mouse models

Matrigel plug mouse model (*n* = 7) and HLI mice (*n* = 8) were injected in the lateral caudal vein with [^68^Ga]Ga-AP747 (5.66 ± 0.57 MBq) and 11 h later with [^68^Ga]Ga-RGD_2_ (5.96 ± 0.95 MBq) on days 1, 3, 7, 10, 13, and 21 after ischemia (Fig. 1). Static PET images were acquired 1 h after intravenous injection of [^68^Ga]Ga-AP747 or [^68^Ga]Ga-RGD_2_ and followed by a CT. Small animal PET imaging acquisition lasted 20 min (numbers of iterations: 4, coincidence: 1–3) using a field of view (FOV) of 10 cm. CT parameters were fixed at 35 kV voltage, 300 ms exposure at medium zoom, acquired by semi-circular method on the same FOV as PET. CT attenuation-corrected reconstruction was performed using Nucline software (Mediso, Budapest, Hungary). Tissue uptake values were expressed as a target-to-background (Matrigel-to-muscle) or as an ischemic-to-contralateral hindlimb PET signal ratio.

### Immunofluorescence of APJ receptor in hindlimb ischemia tissue

After cervical dislocation of HLI mice, the skin of the hind limbs was gently removed, and the tendons were cut. The left (contralateral) and right (ischemic) gastrocnemius muscles were isolated and snap frozen in OCT with isopentane and liquid nitrogen, and 10 µm slices were realized using a cryostat and kept at − 80 °C. The slices were incubated in cold methanol (− 20 °C) for 5 min at RT, washed three times with PBS, and incubated in PBS with 10% of fetal bovine serum and 3% BSA for one hour. Primary anti-APJ antibody (rabbit monoclonal 5H5L9, 2 µg/mL in PBS and 3% BSA, Invitrogen, Waltham, USA) was incubated on the slices overnight at 4 °C in dark wet chamber. Five PBS washes were then realized and a secondary goat anti-rabbit antibody (Alexa fluor 488, 1/500 in PBS and 3% BSA, ThermoFisher, Waltham, USA) was added during 1 h in dark wet chamber. Slides were finally washed three times with PBS +/+ in the dark. Mounting medium (Fluoromount, Invitrogen, Waltham, USA) was added on histological sections. After overnight drying at 4 °C, slides were observed using a NIE microscope (Nikon, Tokyo, Japan) and analyzed using the NIS Elements Imaging software (Nikon, Tokyo, Japan).

### Statistics

Statistical analyses were performed using Prism v9 (GraphPad, San Diego, USA), *P* ≤ 0.05 indicating statistical significance. Autoradiography results were submitted to an unpaired *t *test after checking the data for normal distribution with a Shapiro–Wilk test. In vivo specificity of [^68^Ga]Ga-AP747 PET signal results were submitted to a paired *t* test after checking the data for distribution normality with a Shapiro–Wilk test. Perfusion quantification data in HLI mouse model were compared using a one-way ANOVA test followed by a post hoc Tukey’s multiple comparisons test. PET quantification data of the in vivo longitudinal study were compared using a two-way ANOVA followed by a Sidak’s multiple comparisons post hoc test. Correlations were tested using the Pearson R correlation test.

## Results

### AP747 radiolabeling with gallium-68 leads to excellent and stable radiochemical purity

**Fig. 2:**
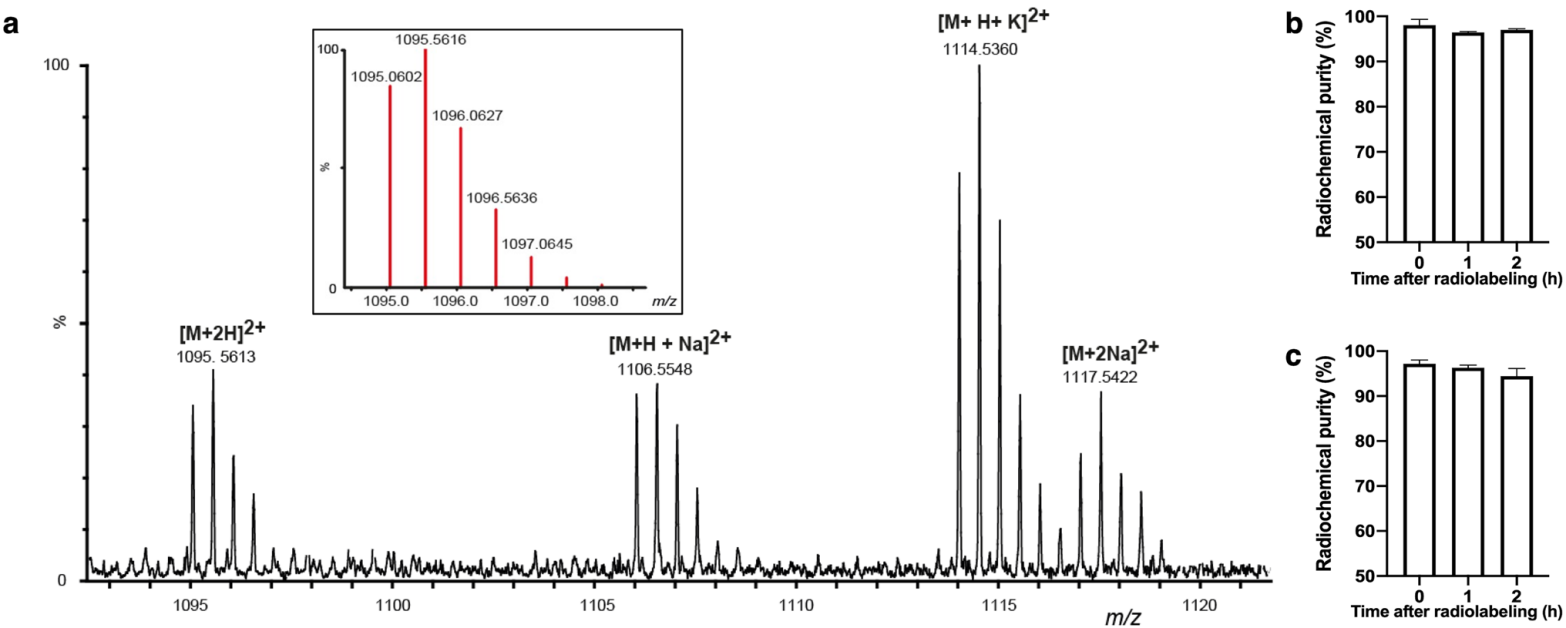
ESI (+) MS spectrum showing the different doubly charged adducts of AP747 (insert: calculated isotopic pattern of [M + 2H]2 + species) (**a**). [^68^ Ga]Ga-AP747 radiochemical purity and stability in physiological serum (**b**) and in human serum (**c**) for two hours

ESI (+)—HRMS spectrum recorded on AP747 showed the formation of several double-charged species corresponding to a chemical composition expected for two NOGADA conjugated on apelin-F13A chain, C_93_H_153_N_29_O_30_S (Fig. 2a). Moreover, the same species were also detected in the presence of different types of cations like sodium and potassium, being both alkalis with high affinity for the NODAGA cages. For the doubly protonated species [M + 2H]^2+^, the experimental value was experimentally measured on the maximum isotopic peak at m/z 1095.5613 (detected C_93_H_155_N_29_O_30_S^2+^; error − 0.3 ppm). Theoretical molecular weight of AP747 was 1830 g/mol with one NODAGA or 2187 g/mol with two NODAGA chelators. AP747 radiolabeling led to a high RCP with gallium-68 (98.1 ± 1.3%, *n* = 3) and with gallium-67 (95.9 ± 0.6%, *n* = 3). RCP of [^68^Ga]Ga-AP747 remained higher than 95% in physiological serum (Fig. 2b) and in human serum (Fig. 2c) up to 2 h after radiolabeling (*n* = 3). NODAGA-RGD_2_ was successfully radiolabeled with gallium-68 (RCP > 95%).

### Radiolabeled AP747 maintains apelin-F13A affinity and specificity for APJ in vitro and in vivo

**Fig. 3:**
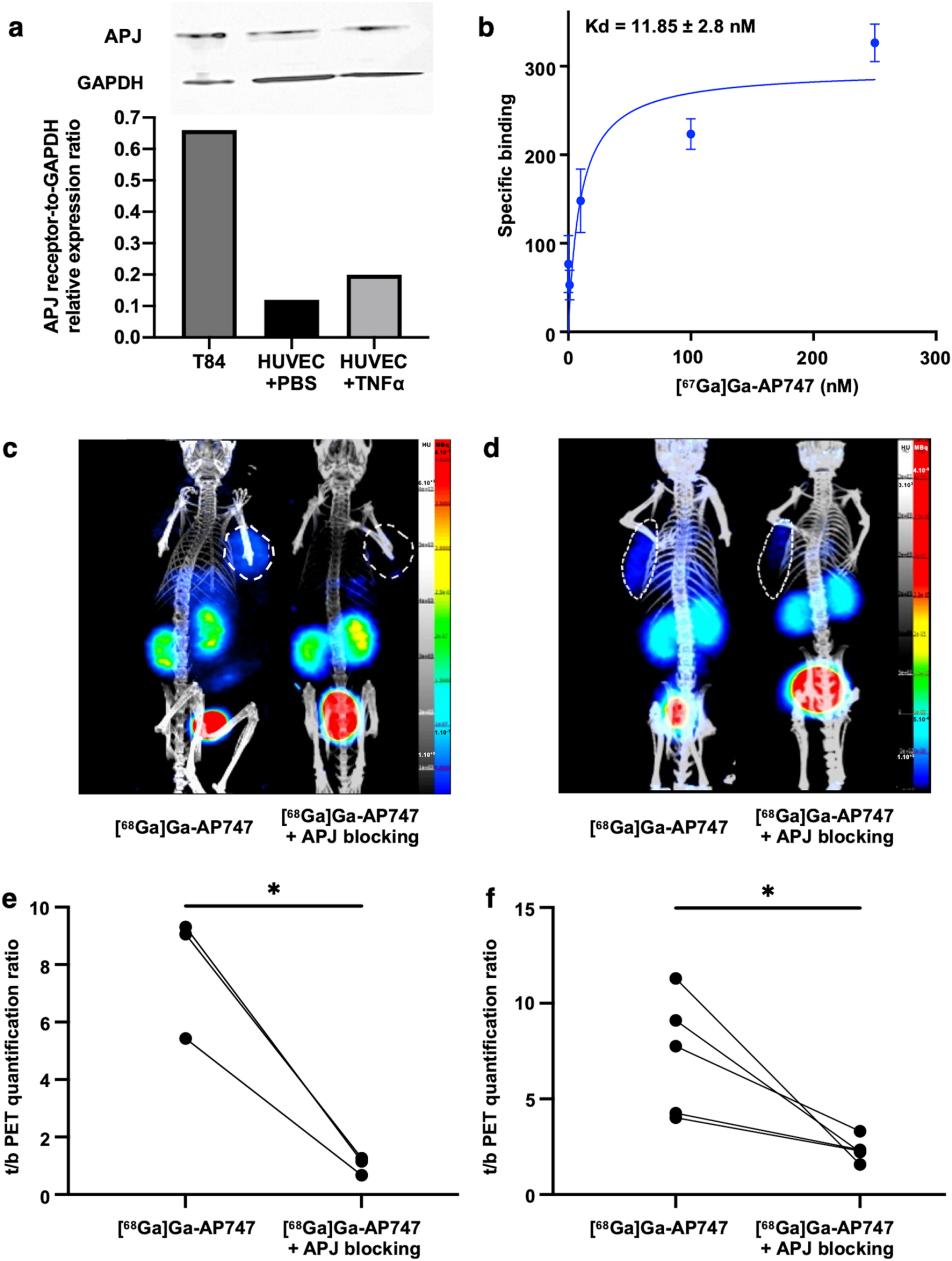
Semi-quantifications of APJ-to-GAPDH expression ratios by Western blot on human adenocarcinoma T84 cells and HUVECs with or without TNF-alpha activation (**a**). Saturation binding curve of [^67^Ga]Ga-AP747 towards APJ receptor on T84 cells (**b**). Representative maximum intensity projections (MIP) images of small animal [^68^Ga]Ga-AP747 PET/CT in an ectopic mice model of human colon adenocarcinoma, in baseline or APJ-blocking conditions (**c**). Representative MIP of small animal [^68^Ga]Ga-AP747 PET/CT images of in a Matrigel plug mice model, in baseline or APJ-blocking conditions (**d**). [^68^Ga]Ga-AP747 PET signal quantifications in a mouse model of ectopic human colon adenocarcinoma in baseline or APJ-blocking conditions (*n* = 3) (**e**). [^68^Ga]Ga-AP747 PET signal quantifications in a Matrigel plug mouse model in baseline or APJ-blocking conditions (*n* = 5) (**f**). c/b standing for cell-to-background; t/b standing for tumor-to-background, mean. *P ≤ 0.05; **P ≤ 0.01; ***P ≤ *0.001*

T84 cells line showed the highest APJ expression. Activated HUVECs showed a higher level of APJ than HUVECs in baseline conditions (Fig. 3a). Saturation binding curves of [^67^Ga]Ga-AP747 on T84 cells revealed a K_d_ value of 11.8 ± 2.8 nM (*n* = 3) (Fig. 3b).

A large excess of 100X non-radiolabeled apelin ligand enabled a highly significant 65.2 ± 11.7% reduction of [^68^Ga]Ga-AP747 binding to T84 cells compared to non-blocked conditions (cell-to-background [^68^Ga]Ga-AP747 autoradiography signal ratios: 0.50 ± 0.23 and 1.40 ± 0.38, respectively, ***P = 0.0006, *n* = 6; Shapiro–Wilk normality tests: [^68^Ga]Ga-AP747 group: *P* = 0.2110; [^68^Ga]Ga-AP747 “blocking” group: *P* = 0.3476). In vivo*,* a large excess (100X) of non-radiolabeled apelin ligand enabled a significant 87.1 ± 0.9% reduction of [^68^Ga]Ga-AP747 PET tumor-to-muscle ratios compared to non-blocked conditions in T84 tumors (1.02 ± 0.31 and 7.93 ± 2.17, respectively, *P = 0.023, *n* = 3; Shapiro–Wilk normality tests: [^68^Ga]Ga-AP747 group: *P* = 0.1100; [^68^Ga]Ga-AP747 “blocking” group: *P* = 0.3365) (Fig. 3c, e). A significant 62.4 ± 21.6% reduction of [^68^Ga]Ga-AP747 PET Matrigel-to-muscle ratios signal was observed compared to non-blocked conditions in Matrigel mice (2.35 ± 0.62 and 7.29 ± 3.14, respectively, *P = 0.031, *n* = 5; Shapiro–Wilk normality tests: [^68^Ga]Ga-AP747 group: *P* = 0.4950, ([^68^Ga]Ga-AP747 “blocking” group: *P* = 0.4264) (Fig. 3d, f).

### [^68^Ga]Ga-AP747 displays a favorable pharmacokinetic profile for PET imaging

**Fig. 4:**
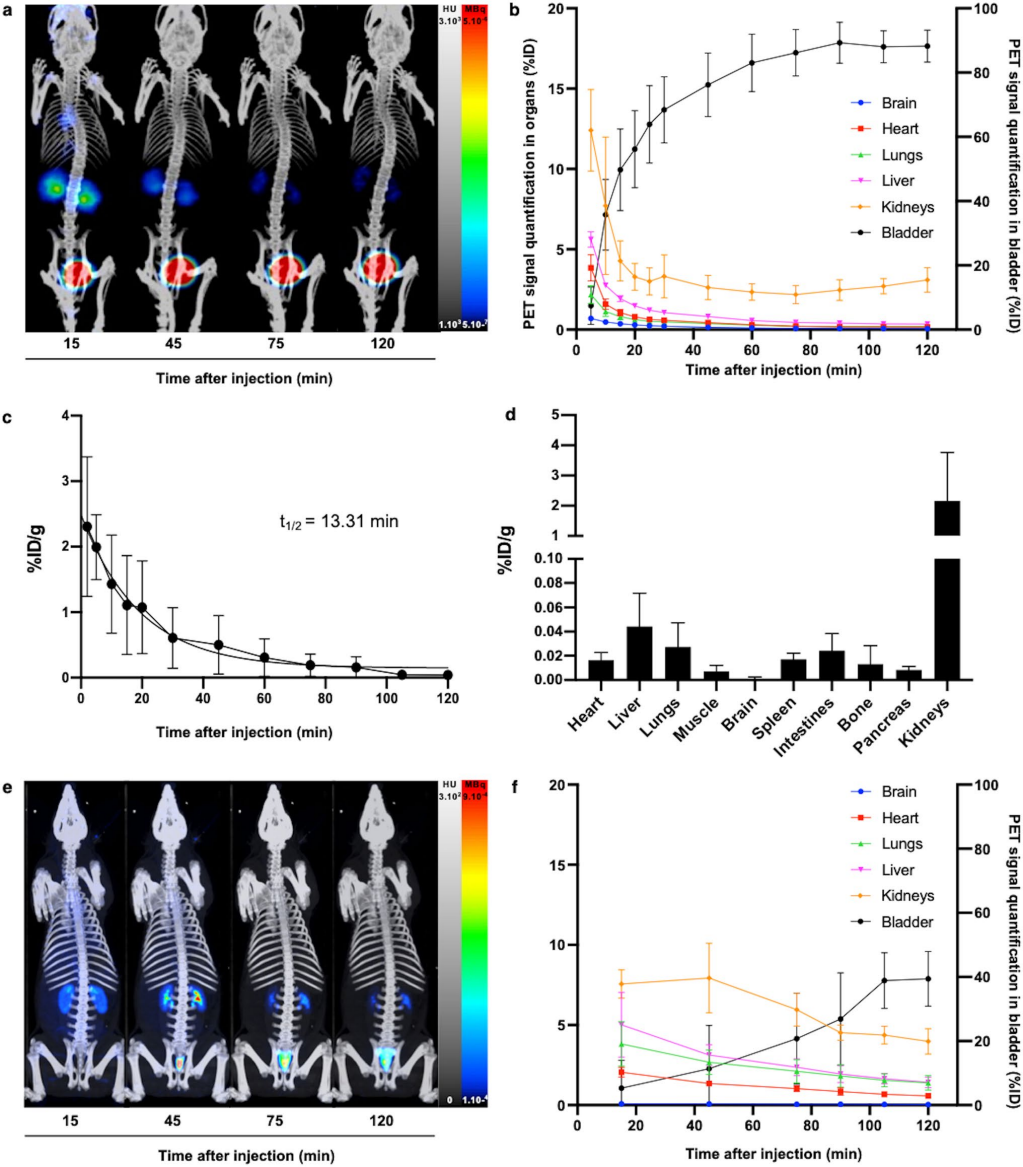
Representative MIP PET images of [^68^Ga]Ga-AP747 biodistribution (**a**) and associated organ quantifications (**b**) in healthy mice (*n* = 3). Blood kinetics of [^68^Ga]Ga-AP747 during 120 min (**c**) and ex vivo gamma counting at 120 min post-injection (**d**) in healthy mice (*n* = 3). Representative [^68^Ga]Ga-AP747 biodistribution MIP PET images (**e**) and associated organ PET quantifications (**f**) in healthy swine (n = 3)

In healthy mice (*n* = 3), the highest [^68^Ga]Ga-AP747 dynamic small animal PET signal was quantified at 120 min in the bladder (88.3 ± 5.0%ID) and in the kidneys (1.04 ± 0.2%ID) without noticeable accumulation in the liver (0.35 ± 0.03%ID), lungs (0.06 ± 0.02%ID), or brain (0.02 ± 0.001%ID) (Fig. 4a, b). Ex vivo gamma counting confirmed these results (Fig. 4d). Plasmatic half-life of [^68^Ga]Ga-AP747 was estimated at 13.3 min (Fig. 4c). In healthy swine (*n* = 3), the highest [^68^Ga]Ga-AP747 PET signal was quantified at 120 min in the bladder (39.43 ± 8.54%ID) and in the kidneys (3.98 ± 0.79%ID) with low accumulation in the liver (1.42 ± 0.34%ID), lungs (1.39 ± 0.46%ID), heart (0.58 ± 0.16%ID), and brain (0.037 ± 0.015%ID) (Fig. 4e, f).

### [^68^Ga]Ga-AP747 small animal PET/CT imaging outperforms [^68^Ga]Ga-RGD_2_ in hypoxic and ischemic models

**Fig. 5:**
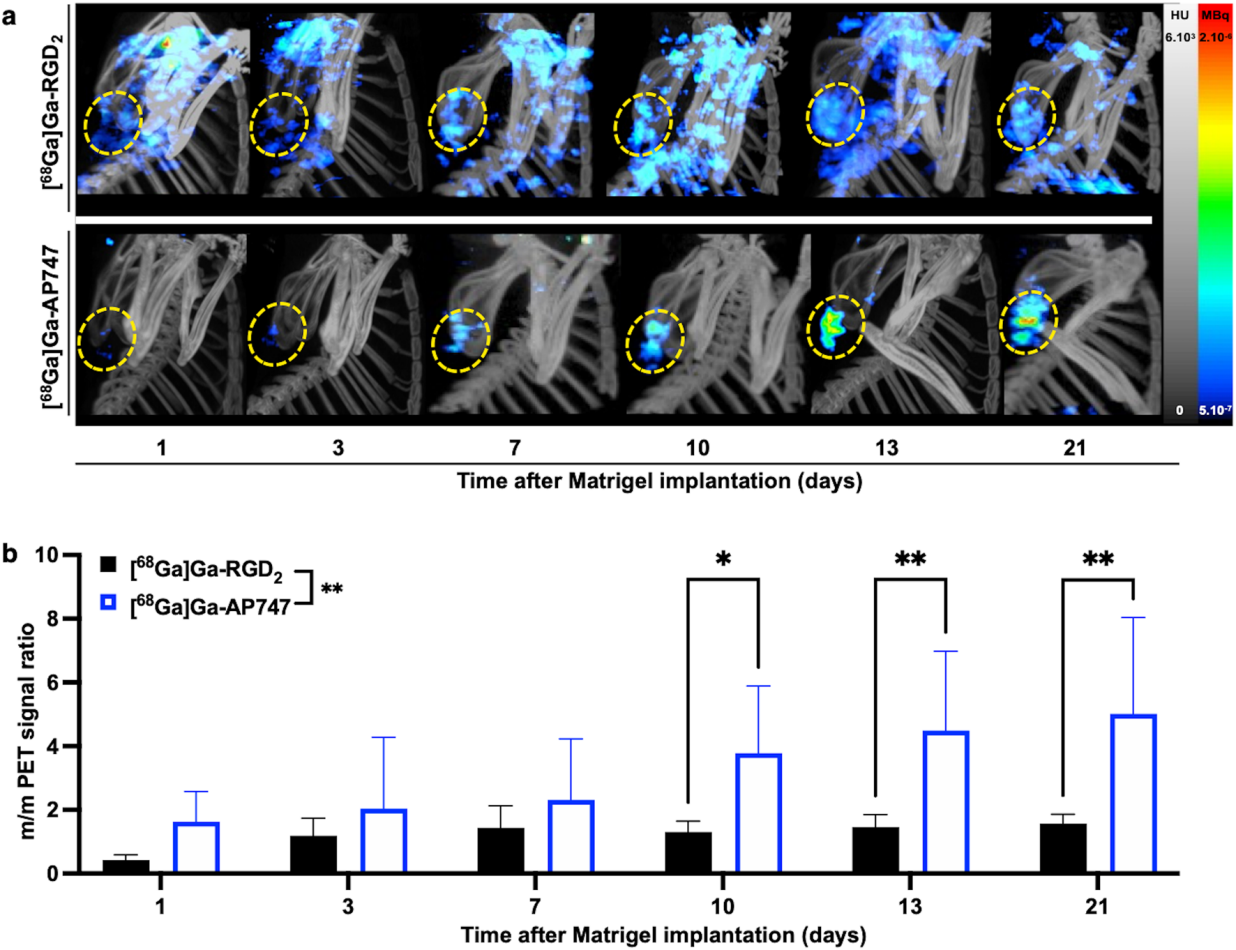
Representative [^68^Ga]Ga-RGD_2_ PET and [^68^Ga]Ga-AP747 MIP PET images (**a**) and related quantifications (**b**) in the Matrigel mouse model over 21 days (*n* = 7). m/m standing for Matrigel-to-muscle. *P ≤ 0.05; **P ≤ *0.01*

In Matrigel mice from day 1 to day 21, the growing target-to-background [^68^Ga]Ga-AP747 small animal PET signal was significantly higher than that of [^68^Ga]Ga-RGD_2_ (**P = 0.0005): 3.78 ± 2.11 and 1.31 ± 0.35, respectively, on day 10 (*P = 0.0362, *n* = 7); 4.86 ± 2.49 and 1.46 ± 0.39, respectively, on day 13 (**P = 0.0064, *n* = 7), 5.00 ± 3.04 and 1.57 ± 0.29, respectively, on day 21 (**P = 0.0016, *n* = 7) (Fig. 5a, b).

**Fig. 6:**
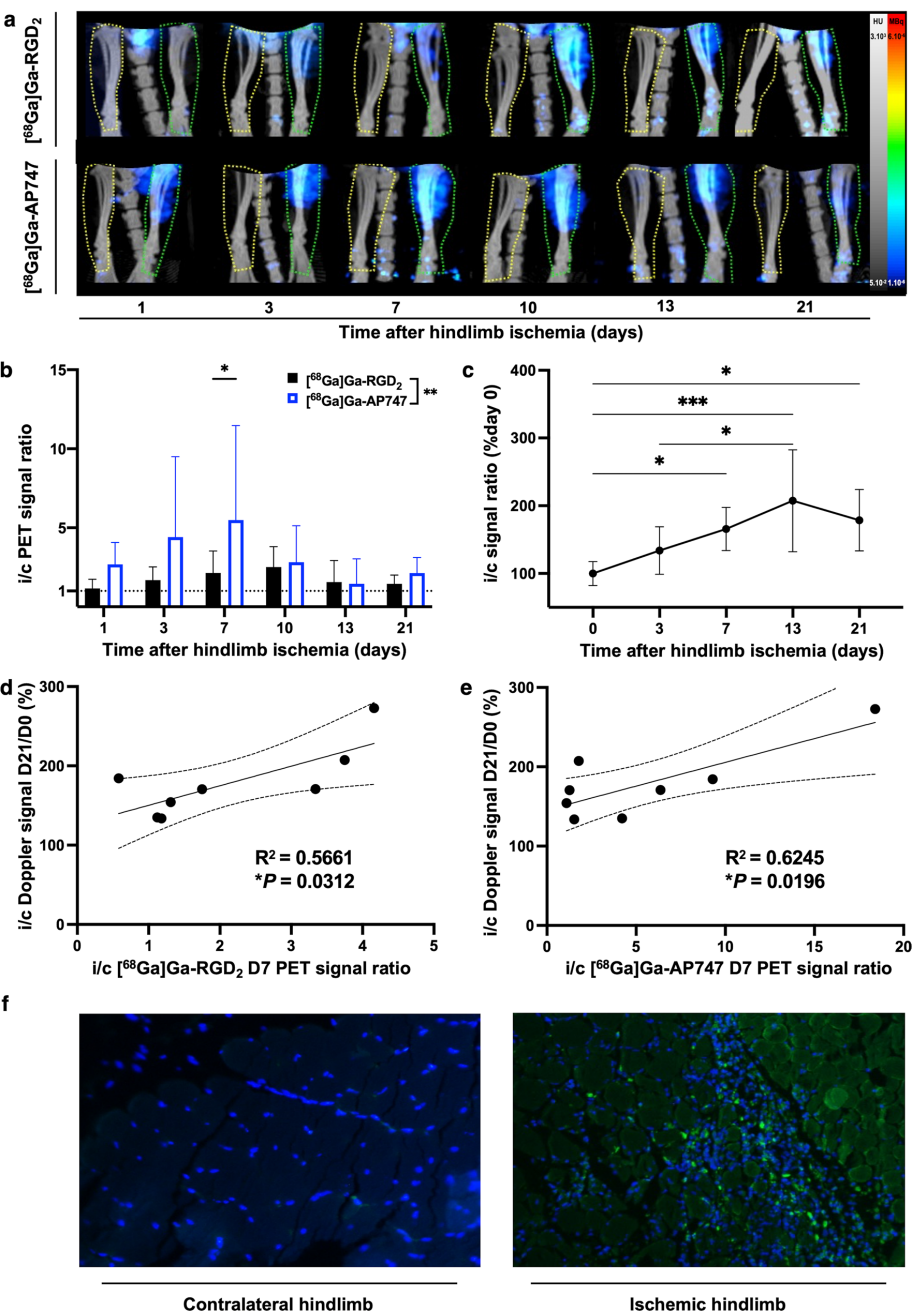
[^68^Ga]Ga-RGD_2_ and [^68^Ga]Ga-AP747 in a hindlimb ischemia model representative MIP PET images **a** [^68^Ga]Ga-RGD_2_ small animal PET/CT and [^68^ Ga]Ga-AP747 small animal PET/CT quantifications (*n* = 8) **b**. LASER Doppler quantifications of hindlimb blood perfusion **c** and correlation studies (**d**, **e**). Representative images of anti-APJ immunofluorescence on day 7 in contralateral hindlimb and ischemic hindlimb of an HLI mouse (**f**). i/c standing for ipsi-to-contralateral. D standing for day. *P < *0.05*

In HLI mice from day 1 to day 21, the i/c [^68^Ga]Ga-AP747 small animal PET signal ratio was significantly higher than that of [^68^Ga]Ga-RGD_2_ (**P = 0.006), and especially on day 7 (respectively, 5.49 ± 5.97 and 2.15 ± 1.38, *P = 0.049, *n* = 8) (Fig. 6a, b). Significant differences were observed in i/c Doppler signal ratios, between days 0 and 7 (respectively, 100.0 ± 17.7% and 165.8 ± 32%, *P = 0.0497), between days 0 and 13 (respectively, 100.0 ± 17.7% and 207.4 ± 75.2%, ***P = 0.0003), between days 0 and 21 (respectively, 100.0 ± 17.7% and 178.6 ± 45.3%, *P = 0.0115) and between days 3 and 13 (respectively, 133.9 ± 35.2% and 207.4 ± 75.2%, *P = 0.0205) (Fig. 6c). [^68^Ga]Ga-AP747 small animal PET signal in the ischemic limb on day 7 significantly and positively correlated with delayed hindlimb perfusion recovery assessed by LASER Doppler on day 21 (*P = 0.0196, *R*^2^ = 0.6245, Fig. 6d), as [^68^Ga]Ga-RGD_2_ on day 7 (*P = 0.0312, *R*^2^ = 0.5661, Fig. 6e). Immunofluorescence confirmed the intense APJ overexpression in ischemic hindlimb tissues on day 7 compared to the contralateral hindlimb (Fig. 6f).

## Discussion

Identification of new targets reflecting the molecular and/or metabolic status of tissues is a major challenge for research and development in molecular imaging. APJ is not only a promising diagnostic biomarker but also a valuable therapeutic target as a key component in numerous pathological contexts, underlying the potential added value of following and monitoring APJ tissue expression. In this study, a novel radiotracer targeting the APJ receptor was developed: [^68^Ga]Ga-AP747, and evaluated for monitoring APJ expression in angiogenic processes at the preclinical stage. Among all the existing APJ agonists and antagonists [33–36], apelin-F13A was chosen for showing one of the best affinities for APJ [41]. The setup of the radiosynthesis led to gallium-68-radiolabeled AP747. Despite the conjugation with NODAGA-chelating agent and subsequent radiolabeling, the affinity of gallium-radiolabeled AP747 for APJ was excellent (11.8 ± 2.8 nM). Colon adenocarcinoma T84 cells and the ensuing xenograft mouse model were chosen because of their high APJ overexpression [30, 42, 43] as confirmed in this study by Western blot. Besides, the in vitro and in vivo targeting specificity of [^68^ Ga]Ga-AP747 confirmed the ability of the radiotracer to bind specifically to APJ. In vivo experiments in healthy mice and swine showed fast urinary elimination of [^68^Ga]Ga-AP747 with low background signal in healthy organs, especially in the liver, resulting in a suitable pharmacokinetic profile for PET imaging. Moving forward to in vivo evaluation on pathophysiological models, APJ expression was first quantified with [^68^Ga]Ga-AP747 small animal PET/CT imaging in a hypoxic model (subcutaneous Matrigel plug mouse model) and then in a hypoxic-ischemic model (hindlimb ischemia mouse model). In both models, [^68^Ga]Ga-AP747 PET signal significantly outbroke [^68^Ga]Ga-RGD_2_ PET signal in terms of target-to-background ratio, a key parameter for PET imaging, but also in terms of signal earliness and intensity. In the Matrigel model, [^68^Ga]Ga-AP747 PET signal progressively increased, probably related to avascular and acellular contents of Matrigel. Indeed, a minimum of ten days is classically required to observe new vessel formation in this model, and a couple of supplementary days to obtain functional vessels [43]. In the hypoxic-ischemic model, a [^68^Ga]Ga-AP747 PET signal peak was observed on day 7 post-ischemia followed by a decrease probably linked to vascular development from popliteal anastomosis and subsequent down-regulation of angiogenesis once the new vessels were formed and functional [44]. Most interestingly, the [^68^Ga]Ga-AP747 PET signal peaking on day 7 significantly and positively correlated with late reperfusion on day 21. Therefore, [^68^Ga]Ga-AP747 could represent a valuable tool for early predictive imaging of tissue reperfusion.

APJ modulation for therapeutic purposes has already been described in the literature [44, 45]. In ischemic therapeutic studies with apelin-13 supplementation were tried, with injections mostly realized before reperfusion [44] or just after ischemia during the 20 first minutes of reperfusion [45], or directly injected on ischemia dermis site just after the reperfusion [46]. These different studies put forward the ability of apelin-13 to reduce damages of ischemia with reduction of oxidative stress and promotion of angiogenesis resulting in protective effects on tissues. Apelin being upregulated after ischemia until 12 h after and with a maximum of expression at 4 h [46]. Our [^68^Ga]Ga-AP747 PET results showed that APJ was overexpressed longer than a week after ischemia, suggesting that a prolonged apelin-13 supplementation could be advantageous for post-ischemic hindlimb perfusion recovery. Such a modulation on APJ expression could be monitored using [^68^Ga]Ga-AP747 PET as companion diagnostic tool, in a broader theragnostic strategy.

## Conclusion

[^68^Ga]Ga-AP747 development enabled PET imaging of APJ expression, constituting an innovative radiotracer for molecular imaging of angiogenesis. [^68^Ga]Ga-AP747 also represents a potent tool to determine therapeutic eligibility to apelin-based therapeutic strategies as a prognostic or diagnostic index like other theragnostic couples in clinical development or routine.

## Abbreviations

^68^Ga: 68-Gallium
CT: Computed tomography
Apelin-F13A: [Ala13]-Apelin-13
HPLC: High-performance liquid chromatography
PET: Positron-emitted tomography
p.i.: Post-injection
VEGF: Vascular endothelial growth factor
VEGFR: Vascular endothelial growth factor receptor
